# Effects of Web-Based Decision Aid to Support Cervical Cancer Screening Decision Among Young Working Women: A Pilot Randomised Controlled Trial

**DOI:** 10.1007/s12529-024-10344-9

**Published:** 2024-12-30

**Authors:** Dorothy Ngo Sheung Chan, Kai Chow Choi, Winnie Kwok Wei So

**Affiliations:** The Nethersole School of Nursing, The Chinese University of Hong Kong, Hong Kong SAR, China

**Keywords:** Decision support techniques, Early detection of cancer, Papanicolaou test, Human papillomavirus DNA tests, Uterine cervical neoplasms

## Abstract

**Background:**

Young working women who devote most of their time to jobs and household chores may experience conflicts when faced with a cervical cancer screening decision. This study aimed to assess the acceptability and feasibility of a Web-based decision aid on cervical cancer screening by young working women, and to preliminarily examine the effects of the decision aid on the knowledge level, risk perception, decisional conflicts, screening decision and screening uptake.

**Method:**

This was a pilot randomised controlled trial. A total of 158 working women aged 25–44 years who had not undergone cervical cancer screening in the past 3 years were recruited. The recruited women allocated to the intervention group received a Web-based decision aid with information about the Papanicolaou test and HPV test, whereas the control group received usual care, i.e. a fact sheet about a healthy living.

**Results:**

A total of 138 of them (72 in the intervention group and 66 in the control group) completed the study and the screening uptake assessment. The intervention group showed greater improvement in the subscale scores and the overall scores for decisional conflicts (effect size, 0.71–0.90), and a statistical significantly larger proportion of the intervention group had undergone cervical cancer screening when compared to the control group (31.9% vs 6.1%). Most interviewees were satisfied with the intervention.

**Conclusion:**

It was feasible and acceptable to implement the Web-based decision aid to young working women. The preliminary findings suggest that the decision aid could help to reduce decisional conflicts and encourage uptake of screening. Full-scale studies are needed to confirm these findings.

**Trial Registration:**

This study was registered at the International Standard Randomised Controlled Trial Number Registry (ISRCTN59163820) on 4 August 2022.

**Supplementary Information:**

The online version contains supplementary material available at 10.1007/s12529-024-10344-9.

## Introduction

Cervical cancer is the fourth most common cancer and the fourth-leading cause of cancer-related deaths among women worldwide. Its global incidence and associated mortality rates among women aged ≥ 25 were 25.3 and 13.9 per 100,000 population, respectively, in 2022 [1]. The risk of developing cervical cancer can be reduced by undergoing routine screening tests, and the Papanicolaou (Pap) test and HPV test are those that are most used [2]. Population-based cervical cancer screening programmes had been launched in various countries and regions such as the USA and UK. For example, population-based and voluntary cervical cancer screening programme was launched in Hong Kong in 2004 and it is recommended that asymptomatic women aged 25–64 with a history of sexual activity should consider undergoing screening comprising two annual Pap tests, followed by a Pap test every 3 years [2, 3]. Besides, recently an HPV test has been recommended as an alternative to a Pap test, and women aged 30–64 are recommended to undergo either an HPV test or an HPV test plus a Pap test every 5 years [2].

Despite the effectiveness of screening in reducing the incidence of cervical cancer, previous surveys revealed that the screening uptake rate varies across countries/regions [4–6]. For example, the screening uptake rate was found to be only 52.1% in the Population Health Survey 2020–2022 conducted in Hong Kong, which was lower than the uptake rates in the USA and UK [4–6]. Particularly, the uptake rates among women aged 25–34 and 35–44 were 29.2% and 57.3%, respectively, in Hong Kong, whereas the uptake rate among women aged 25–44 in the USA was reported to be 84.5% [4, 5]. A survey conducted in Australia also revealed that more younger women were due, overdue or had never had screening than their older counterparts [7].

Women’s decisions on whether to undergo cervical cancer screening may depend on several factors that influence their behaviours. For example, factors such as age, marital status, knowledge of the disease and screening, risk perceptions, perceived benefits of and barriers to screening, and cultural views and values considerably affect women’s decisions on screening [8, 9]. Women of younger age are of childbearing age and may be employed and/or married. Women tend to avoid undergoing screening due to competing demand for time due to employment and family responsibilities [7, 10]. This suggests that younger women who have dual roles (juggle with jobs and family responsibilities) may have more factors to consider when making a screening decision.

To encourage women to pursue cervical cancer screening, appropriate interventions are needed that address their concerns and enhance screening uptake. Studies have revealed that interventions with content that highlights the importance of cervical cancer screening, can effectively enhance women’s knowledge and health beliefs, hence enhancing the screening uptake [11, 12]. However sometimes, improved knowledge and beliefs do not always guarantee an uptake of screening; related issues such as decisional conflicts in the presence of several screening options and expectations can affect a person’s decision [12]. A Cochrane review suggested that decision aids could be a useful tool for assisting people to make well-informed decisions about undergo screening for a disease [12]. Such aids are designed to provide information about screening options that are available and outcomes that are relevant to a person’s health status. Features of decision aids that increase their utility are details on the benefits and risks of undergoing screening, a values-clarification exercise and guidance on decision-making [12, 13]. As such, decision aids are effective in helping people make screening decisions, reducing their decisional conflicts and clarifying their risk perceptions and values [12].

Although decision aids have been shown to be useful in resolving decisional conflicts and enhancing screening decision, currently, decision aids for colorectal, breast and prostate cancer screening are available, and only a limited number are designed for cervical cancer screening [14–16]. Furthermore, most decision aids are paper-based, and only a few are Web-based [12, 14]. However, rapid technological advances have increased people’s utilisation of Internet-capable electronic devices, such as computers and smartphones, to browse the Web and receive up-to-date information [17]. People have traditionally received health information from talks, television broadcasts, pamphlets or face-to-face counselling [14, 16]. However, health talks and counselling are not always held across an entire country/region, and their timings may not match everybody’s schedule, especially those of women who are engaged in part-time or full-time jobs. This indicates that the dissemination of health information via the Web could overcome geographic inaccessibility and thereby ensure that more people are informed on health matters than via non-Web-based means. This would be particularly useful for young working women, as they would be able to access the information at any time or place of their convenience. This study aimed to test the feasibility and acceptability of a Web-based decision aid for decision-making on cervical cancer screening in working women aged 25–44. This study also aimed to preliminarily examine its effects on working women’s knowledge level, risk perception, decisional conflicts, screening decision and screening uptake. The objectives of the study were to (1) assess the acceptability, feasibility and uptake of the Web-based decision aid among working women aged 25–44 years; (2) determine an initial estimate of the effectiveness of the decision aid in reducing decisional conflicts concerning cervical cancer screening among the study population; (3) determine an initial estimate of the effectiveness of the decision aid in improving knowledge on cervical cancer and screening and risk perceptions about the disease among the study population and (4) determine an initial estimate of the effectiveness of the decision aid in screening decisions and actual screening uptake among the study population.

## Methods

### Study Design

This study was conducted from October 2022 to September 2023 and comprised two phases, namely the development and validation of the Web-based decision aid, and a pilot randomised controlled trial (RCT) consisting of a pre-test, a post-test and a 3-month follow-up.

### Phase One: Development and Validation of the Web-Based Decision Aid

The decision aid was developed based on the Ottawa Decision Support Framework [18], which assesses the following three aspects: decisional needs, decision support and decision outcome. Decisional needs are those concerning a person’s decisional conflicts, knowledge and expectations, personal values and the nature of the decision. ‘Decision support’ refers to interventions that can be delivered through clinical counselling, decision aids and decision coaching. Decision support interventions like decision aids assist users by helping them to address their decisional needs and clarify their values and supplying them with the facts on and probable outcomes of their chosen health options. Decision outcome is dictated by the facts upon which a decision is based and a person’s value-based preferences after they receive decision support [18]. Based on individual decisional needs, the decision support intervention provides relevant information to address specific concerns. The effects of the decision support intervention were then evaluated to reveal its impact on decisional outcomes. The Web-based decision aid contained (1) information on the incidence and mortality rates, risk factors, signs and symptoms, treatment options and survival rates for cervical cancer; (2) information on the available screening options, including the Pap test and HPV test; (3) current cervical cancer screening recommendations and a link to the government website that lists clinics that perform screening; (4) information on the similarities of and differences between screening methods, and the accuracy of each method; (5) information on the risks and benefits of screening; (6) a values-clarification exercise and information on time management and (7) structured guidance on decision-making. A question-and-answer section (‘Talk to Nurse’) was also included to allow women to post questions if they needed to seek additional information.

The contents of the decision aid were first drafted. Webpage design and graphic design companies were hired to devise the layout, graphics, typography and content-presentation of the Web-based decision aid. A six-member working group comprising healthcare professionals, representatives of community partners and target users was first established to review the contents and layout of the first draft of the decision aid. Based on the feedback received from the group, the decision aid was revised and then reviewed by a validation panel comprising five experts from health-related backgrounds and 10 young working women. They were invited to comment on the appropriateness and relevance of the decision-aid content items. Based on the panel’s comments on the content and layout of the decision aid, it was revised again, with the revisions largely involving alterations of the layout, such as the font size, the spacing between words and lines, the placement of words, the terms used, the colour scheme and the removal of non-essential static or motion graphics.

### Phase Two: Pilot RCT

#### Participants and Settings

Potential participants were recruited at non-governmental organisations and at places where women gathered, such as workplaces. The inclusion criteria were (1) a Chinese woman aged 25–44 with a Hong Kong Identity Card; (2) engaged in part-time or full-time employment; (3) no history of cervical cancer or total hysterectomy; (4) a history of sexual activity; (5) able to read or communicate in Chinese or Cantonese; (6) undergone no Pap or HPV tests in the past 3 years and (7) have at least one computer, tablet or smartphone. The sample size was estimated based on the main outcome of decisional conflict. A meta-analysis of 35 studies that have assessed the effects of decision aids on people facing treatment or screening decisions reported a pooled mean difference of − 7.81 (95% confidence interval [CI], − 9.84 to − 5.77) or, equivalently, a pooled standardised mean difference (SMD) of − 0.45 (95% CI, − 0.56 to − 0.34) for a total decisional conflict score (primary outcome) [12]. It was estimated by using G*Power 3.1 that a sample size of 158 participants with 79 per arm in a two-parallel-arm RCT would give 80% power to detect an effect size of 0.45 SMD for decisional conflict after the intervention, at a 5% level of significance (two-sided). The study was an exploratory trial and mainly conducted to examine the acceptability, feasibility of a Web-based decision aid and preliminarily estimate its effects. As statistical power was not the main concern at this stage, no further allowance for attrition rate was considered.

#### Randomisation and Blinding

Block randomisation with a block size of eight in all but the last block was used to allocate eligible participants to the intervention group or the control group in a 1:1 ratio. Randomisation was performed by a statistician using a computer-generated random schedule. A research assistant was responsible for participant recruitment and was blinded to the participants’ group allocation. The group assignments were sealed in sequentially numbered, opaque envelopes that were opened after the participants were screened for eligibility, had signed the consent form and had completed the baseline questionnaire.

#### Intervention and Control Groups

Trained research nurses delivered the intervention by sharing the link of the Web-based decision aid with the intervention group. The link was shared through (1) a printed QR code that was distributed to the participants with instructions to scan the QR code using their tablet or mobile phone or (2) email or WhatsApp messages to the participants. The research nurses demonstrated how to use the decision aid on a tablet or a computer or mobile phone with internet access. The nurses briefly explained how to browse the decision aid using the icons for the content included in the decision aid, and the intervention group then read the decision aid in their own time in a convenient location. Participants were able to browse the decision aid whenever and wherever they wanted. In view of their employment status, the participants were advised to read the decision aid daily in a sequential manner. They were contacted a week later to determine whether they had used or encountered difficulty in using the decision aid. The control group received the minimal information control.

#### Outcome Measures

The outcome measures assessed (1) decisional conflicts; (2) knowledge about cervical cancer screening; (3) risk perception; (4) screening decision and (5) screening uptake. Socio-demographic data were collected at baseline (T0). Information on decisional conflicts, knowledge, risk perception and screening decision was collected at T0 and 2 weeks after the intervention (T1). Screening uptake was collected 3 months after the intervention (T2).

Decisional conflicts were measured using the 16-item Decisional Conflict Scale (DCS), which comprises five subscales, namely the Informed, Values Clarity, Support, Uncertainty and Effective Decision subscales [19]. Each item is scored from 0 (‘Strongly agree’) to 4 (‘Strongly disagree’). The total scores of the subscales are converted to a score out of 100, and a total score less than or equal to 25 indicates that a person has low decisional conflict, whereas a total score equal to or greater than 37.5 indicates that a person has decisional delay [19]. Knowledge about cervical cancer screening was assessed using an 11-item tool [20, 21] in which each item is answered with ‘Agree’, ‘Disagree’ or ‘Unsure’. One point is awarded for each correct answer, zero points are awarded for each incorrect or ‘unsure’ answer, and a high total score indicates a high level of knowledge. Risk perception was assessed by asking the participants whether their perception of their risk of developing cervical cancer was low, moderate or high. Screening decision was assessed by asking the participants a choice predisposition question and a choice question. The choice predisposition question assessed the participants’ inclination towards an option and was rated on a 15-point scale ranging from 1 (an inclination towards answering ‘yes’ in response to an option) to 15 (an inclination towards answering ‘no’ in response to an option). A sample question was ‘If you are asked to make a choice about a Pap test, please show your inclination to undergo one on a scale of 1 to 15’. The choice question assessed whether the participants would discuss their screening decision with a doctor and a ‘No/Yes/Unsure’ format to elicit participants’ decision on the use of a particular screening option (Pap test/HPV test) [22]. A sample question was ‘Now that you have had a chance to talk to your doctor about having Pap test, which choice (1. Not having Pap test, 2. Having a Pap test, 3. Unsure) looks the best for you?’ Screening uptake was reported by the participants as ‘Yes’ or ‘No’, based on whether they had undergone a Pap test and/or an HPV test, as verified by presenting their test receipt.

The acceptability and feasibility of the intervention were assessed after it had been delivered, via a semi-structured telephone interview with 20 members of the intervention group. They were asked to give their general impressions of using the decision aid; to comment on the clarity and length of the information presented, and the amount and objectivity of the information; to comment on how much the decision aid guided their decision-making; to comment on when and where they had read the decision aid; to comment on the adequacy of the intervention duration; and to comment on whether the decision aid had influenced their choice to undergo/not undergo screening. Feasibility was assessed by comparing the number of young working women who were approached, screened and subsequently deemed eligible to participate in the study with the number who had been recruited, had completed the intervention and had not dropped out of the study.

#### Data Collection Procedure

Ethical approval was obtained from the Joint CUHK-NTEC Clinical Research Ethics Committee of the Chinese University of Hong Kong (Ref. No.: 2021.700). The research assistant approached potential participants and obtained their written consent after they had been determined to be eligible to participate. The participants were asked to complete a pen and paper questionnaire at T0 and an online questionnaire at T1. They were contacted by telephone at T2 to collect information about their screening uptake. The acceptability of the decision aid was determined by interviewing 20 members of the intervention group via telephone. The interviews lasted approximately 15–30 min, were conducted by the research assistant and were audio-recorded. An incentive (HKD100 = US12.8) was provided to the participants to appreciate their participation in this study.

#### Statistical Analysis

The normality of continuous variables was determined by their skewness and kurtosis statistics and normal probability plots. There was no continuous variable violated normality. Therefore, the data were summarised and presented as means together with standard deviations (for continuous variables) or frequencies with percentages (for categorical variables). The between-group differences of baseline characteristics were assessed using an independent *t*-test (for continuous variables) and a chi-square or Fisher’s exact test (for categorical variables). A generalised estimating equation (GEE) model was used to compare the differential change in each of the continuous outcome scores for decisional conflict, cervical cancer screening knowledge and choice predisposition at T1 relatively to T0 between groups. In particular, a group-by-time interaction term was included in the model to assess the differential change between groups. The categorical outcomes of risk perceptions, screening decision and screening uptake were compared between groups using chi-square or Fisher’s exact tests, as appropriate. The study design was a randomised controlled trial, which was expected to control all observed and unknown confounding factors. To the best of our knowledge, there were no well-known prognostic factors for the study outcomes. Therefore, no adjustments were made for any covariates in the outcome analyses. Hedges’ *g* effect sizes together with 95% CIs were calculated for the continuous outcomes based on the SMDs of the changes in their scores at T1 with respect to T0, while a rate ratio with a 95% CI was calculated for the screening uptake at T2 to determine an initial estimate of the effect sizes of the intervention on the different outcomes. All the statistical analyses were conducted using IBM SPSS 26 (IBM Corp., Armonk, NY) with the level of significance set to 0.05 (two-sided). The recordings of the semi-structured interviews were transcribed verbatim. The data were analysed using content analysis. Content analysis aims to search text for recurring words, provide counts of the recurring words and categorise the content of interview data. The interpretation focuses on objectively identifying and quantifying patterns or relationships within the content. The investigator gains insights into the prevalence of certain topics or messages. Content analysis provides the most suitable method for data analysis given the goal of assessing the intervention groups’ experience of using the decision aid through interviews. The data were coded, and these data-driven codes were collated into themes to represent contextual meaning [23].

## Results

### Feasibility of the Study

**Fig. 1:**
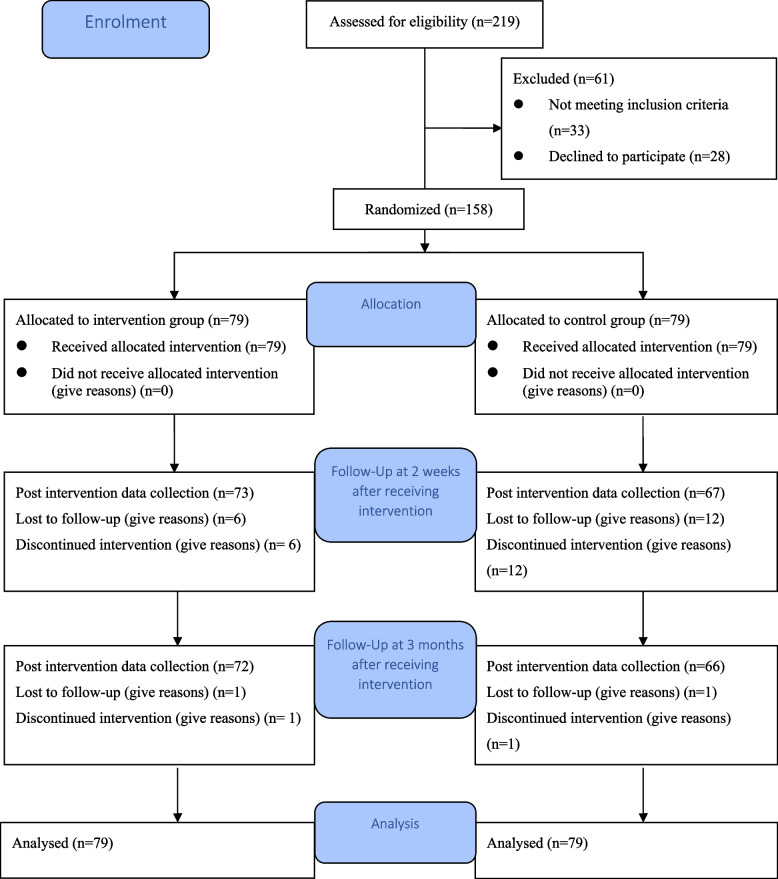
Flow diagram of intervention and data collection points

Two hundred and nineteen potential participants were approached. Thirty-three potential participants were ineligible because of age, employment or sexual activity status, or because they were not due to undergo screening, equating to an eligibility rate of 84.9%. Twenty-eight potential participants declined to participate due to a lack of interest in the topic of cervical cancer and screening or a lack of time due to work and providing childcare. The remaining 158 potential participants consented to participate, equating to a consent rate of 84.9%. The intervention group received the decision aid. Eighteen participants (six from the intervention group and 12 from the control group) did not respond to the follow-up phone call and dropped out at T1. The intervention completion rate of the intervention group was 92.4%. Two participants dropped out at T2. Thus, the overall retention rate was 87.3% (Fig. 1).

### Participants’ Characteristics

The mean age of the participants was 37.1 years (standard deviation = 5.6), and 63% were married. Approximately 41% had received an HPV vaccination. The participants’ characteristics were generally homogeneous across both groups (Table 1).

**Table 1:** Baseline characteristics of the participants (*N* = 158)

Characteristics	Control (*n* = 79)	Intervention (*n* = 79)	*P*
Age (years)^†^	36.7 (5.7)	37.6 (5.5)	0.301^a^
Marital status			
Married	50 (63.3%)	50 (63.3%)	0.999^b^
Single/divorced/separated	29 (36.7%)	29 (36.7%)	
Having children			
No	27 (34.2%)	26 (32.9%)	0.866^b^
Yes	52 (65.8%)	53 (67.1%)	
Educational level			
Secondary school or below	37 (46.8%)	36 (45.6%)	0.507^b^
Matriculation/associate degree/higher diploma	9 (11.4%)	14 (17.7%)	
University or above	33 (41.8%)	29 (36.7%)	
Monthly household income (HK$)			
< 20,000	26 (32.9%)	28 (35.4%)	0.481^b^
20,000– < 40,000	21 (26.6%)	26 (32.9%)	
≥ 40,000	32 (40.5%)	25 (31.6%)	
Family history of cervical cancer			
No	79 (100.0%)	77(97.5%)	0.497^c^
Yes	0 (0.0%)	2 (2.5%)	
Having health insurance			
No	43 (54.4%)	46 (58.2%)	0.630^b^
Yes	36 (45.6%)	33 (41.8%)	
Ever had HPV vaccination			
No	47 (59.5%)	46 (58.2%)	0.872^b^
Yes	32 (40.5%)	33 (41.8%)	

Data marked with ^†^ are presented as mean (standard deviation), all others are presented as frequency (%)

^a^Independent *t*-test; ^b^chi-square test; ^c^Fisher’s exact test

### Effects of the Web-Based Decision Aid on Decisional Conflict, Risk Perceptions, Knowledge and  Decision

At T0, there were no statistically significant between-group differences in overall decisional conflict, risk perception, knowledge or screening decision. The scores on all subscales of decisional conflict were comparable between the groups except for those on the uncertainty subscale (Tables 2, 3, and 4).

**Table 2:** Decisional conflict, knowledge and screening decision (choice predisposition) outcomes across study time points between the control and intervention groups

Outcomes^†^		Control	Intervention	Effect size (95% CI)^#^
Decisional Conflict Scale				
Informed subscale	T0 (*n* = 158)	40.0 (17.3)	42.3 (20.1)	
(Possible score range, 0–100)	T1 (*n* = 140)	37.4 (18.3)	22.5 (17.3)	0.90 (0.55, 1.24)
Values Clarity subscale	T0 (*n* = 158)	36.2 (16.5)	39.8 (21.9)	
(Possible score range, 0–100)	T1 (*n* = 140)	33.3 (16.6)	22.5 (16.5)	0.81 (0.46, 1.15)
Support subscale	T0 (*n* = 158)	37.4 (15.8)	41.8 (18.3)	
(Possible score range, 0–100)	T1 (*n* = 140)	36.4 (17.7)	24.1 (17.0)	0.88 (0.53, 1.22)
Uncertainty subscale	T0 (*n* = 158)	34.7 (16.1)	41.0 (18.7)	
(Possible score range, 0–100)	T1 (*n* = 140)	36.6 (17.6)	25.7 (18.1)	0.83 (0.48, 1.17)
Effective decision subscale	T0 (*n* = 158)	30.0 (14.4)	33.1 (16.9)	
(Possible score range, 0–100)	T1 (*n* = 140)	30.7 (14.1)	21.2 (16.1)	0.71 (0.36, 1.04)
Overall scale	T0 (*n* = 158)	35.3 (13.8)	39.2 (15.8)	
(Possible score range, 0–100)	T1 (*n* = 140)	34.6 (14.5)	23.1 (15.3)	0.99 (0.63, 1.33)
Knowledge about cervical cancer screening				
Knowledge score	T0 (*n* = 158)	7.6 (1.8)	7.4 (1.8)	
(Possible score range, 0–11)	T1 (*n* = 140)	7.9 (1.6)	8.2 (1.9)	0.20 (− 0.14, 0.53)
Screening decision—choice predisposition				
Predisposition towards choosing Pap test*	T0 (*n* = 158)	4.6 (2.7)	4.9 (2.9)	
(Possible score range, 1–15)	T1 (*n* = 140)	5.1 (2.9)	4.7 (3.5)	0.20 (− 0.13, 0.53)
Predisposition towards choosing HPV test*	T0 (*n* = 158)	6.0 (2.9)	6.4 (3.2)	
(Possible score range, 1–15)	T1 (*n* = 140)	5.9 (3.1)	5.5 (3.3)	0.27 (− 0.06, 0.60)

^†^Variables are presented as mean (standard deviation)

^*^Choice predisposition assesses a person’s inclination towards an option and is rated on a 15-point rating scale ranging from 1 (inclination towards ‘yes’ for an option) to 15 (inclination towards ‘no’ for an option)

^#^Hedges’ *g* effect size which corresponds to the standardised mean difference of the mean changes at T1 with respect to T0 between the intervention and control groups

**Table 3:** Risk perceptions and screening decision (choice question) outcomes across study time points between the control and intervention groups

Outcomes^#^	Control	Intervention	*P*
Risk perceptions			
Perceived risk of having cervical cancer at T0			
Low	37 (46.8%)	38 (48.1%)	0.808^a^
Moderate	36 (45.6%)	37 (46.8%)	
High	6 (7.6%)	4 (5.1%)	
Perceived risk of having cervical cancer at T1			
Low	36 (53.7%)	27 (37.0%)	0.041^a^
Moderate	28 (41.8%)	35 (47.9%)	
High	3 (4.5%)	11 (15.1%)	
Screening decision—choice questions			
Screening decision—use of Pap test at T0			
No	4 (5.1%)	0 (0.0%)	0.182^b^
Yes	57 (72.2%)	59 (74.7%)	
Unsure	18 (22.8%)	20 (25.3%)	
Screening decision—use of Pap test at T1			
No	3 (4.5%)	6 (8.2%)	0.090^b^
Yes	45 (67.2%)	57 (78.1%)	
Unsure	19 (28.4%)	10 (13.7%)	
Screening decision—use of HPV test at T0			
No	7 (8.9%)	2 (2.5%)	0.075^b^
Yes	42 (53.2%)	35 (44.3%)	
Unsure	30 (38.0%)	42 (53.2%)	
Screening decision—use of HPV test at T1			
No	3 (4.5%)	5 (6.8%)	0.195^b^
Yes	34 (50.7%)	46 (63.0%)	
Unsure	30 (44.8%)	22 (30.1%)	

^#^Variables are presented as frequency (percentage)

^a^Chi-square test; ^b^Fisher’s exact test

**Table 4:** Generalised estimating equation (GEE) analysis for comparing the decisional conflict, knowledge and choice predisposition outcomes across study time points between the control and intervention groups

Outcomes	Regression coefficients of the GEE models					
	Group		Time		Group × time	
	*β* (95% CI)	*P*	*β* (95% CI)	*P*	*β* (95% CI)	*P*
DCS						
Informed subscale	2.32 (− 3.48, 8.13)	0.433	− 2.15 (− 6.35, 2.04)	0.314	− 17.83 (− 24.60, − 11.05)	< 0.001
Values clarity subscale	3.59 (− 2.43, 9.60)	0.242	− 2.46 (− 5.77, 0.86)	0.146	− 14.96 (− 21.03, − 8.89)	< 0.001
Support subscale	4.33 (− 0.97, 9.62)	0.110	− 0.83 (− 4.92, 3.26)	0.691	− 17.02 (− 23.44, − 10.59)	< 0.001
Uncertainty subscale	6.33 (0.92, 11.74)	0.022	2.09 (− 2.24, 6.42)	0.344	− 17.43 (− 24.35, − 10.50)	< 0.001
Effective decision subscale	3.09 (− 1.79, 7.96)	0.214	0.78 (− 2.75, 4.30)	0.666	− 12.69 (− 18.53, − 6.84)	< 0.001
Overall scale	3.88 (− 0.71, 8.47)	0.098	− 0.40 (− 3.60, 2.80)	0.806	− 15.82 (− 21.13, − 10.51)	< 0.001
CCS knowledge score	− 0.15 (− 0.70, 0.40)	0.589	0.34 (− 0.08, 0.76)	0.110	0.38 (− 0.25, 1.01)	0.234
Choice predisposition						
Predisposition towards choosing Pap test	0.35 (− 0.52, 1.23)	0.427	0.43 (− 0.21, 1.07)	0.189	− 0.71 (− 1.74, 0.32)	0.174
Predisposition towards choosing HPV test	0.43 (− 0.52, 1.38)	0.373	0.00 (− 0.70, 0.70)	0.994	− 0.86 (− 1.95, 0.24)	0.124

*DCS*, Decisional Conflict Scale; *CCS*, cervical cancer screening

Only the model estimates of regression coefficients of the dummy variables for group (group: 0 = control (reference); 1 = intervention), time point (time: 0 = T0, pre-test (reference); 1 = T1, post-test), and group-by-time interaction term (group × time) are shown for each GEE model for each outcome

Between-group comparisons of changes in the subscale and overall scores on the DCS from T0 to T1 revealed that there was generally a statistically significantly greater improvement in the subscales and overall scores in the intervention group than in the control group. The Hedges’ *g* effect sizes of the subscales and overall scores ranged from 0.71 to 0.99 (Table 2). At T1 and compared with the control group, the intervention group exhibited a greater improvement in certainty about the best choice, in feeling informed about the screening options and support, in their clarity about their personal values and in their decision-making effectiveness. The GEE analyses revealed statistically significant group-by-time interaction effects on the Informed (*β* =  − 17.83; 95% CI, − 24.60 to − 11.05; *p* < 0.001), Values Clarity (*β* =  − 14.96; 95% CI, − 21.03 to − 8.89; *p* < 0.001), Support (*β* =  − 17.02; 95% CI, − 23.44 to − 10.59; *p* < 0.001), Uncertainty (*β* =  − 17.43; 95% CI, − 24.35 to − 10.50; *p* < 0.001) and Effective Decision (*β* =  − 12.69; 95% CI, − 18.53 to − 6.84; *p* < 0.001) subscales. At T1 and compared with the control group, the intervention group demonstrated a statistically significantly greater reduction in overall decisional conflict score (*β* =  − 15.82; 95% CI, − 21.13 to − 10.51; *p* < 0.001) (Table 4).

Furthermore, from T0 to T1 and compared with the control group, a statistically significantly larger proportion of the intervention group perceived their risk of developing cervical cancer as high (4.5% vs 15.1%; *p* = 0.041). However, there were no such between-group differences regarding knowledge or screening decision (Tables 2, 3, and 4).

### Effects of the Web-Based Decision Aid on Screening Uptake

At T2 and compared with the control group, a larger proportion of the intervention group had undergone screening (6.1% [4 out of 66] vs 31.9% [23 out of 72], *p* < 0.001) with a rate ratio of 5.27 (95% CI, 1.92 to 14.44). Among the screened participants in the intervention group, 20 had undergone a Pap test, two had undergone an HPV test and one had undergone both tests. Among the screened participants in the control group, three had undergone a Pap test while the remaining one had undergone both tests. Among those who had not undergone screening, common reasons were a lack of time due to busyness at work, regarding it as unnecessary, having not arranged the appointment yet and due to the cost of screening.

### Acceptability of the Intervention

Twenty participants in the intervention group were interviewed, and six of these participants had undergone screening. Four categories were generated regarding the acceptability of and experience in using the decision aid: (1) a clear and user-friendly design; (2) informative and balanced content aiding decision-making; (3) facilitators of using the decision aid and (4) areas for improvement.

1. Clear and user-friendly design

Three-quarters of the interviewees agreed that the Web-based decision aid was user-friendly, with clear and concise content. They thought that the similarities, differences and accuracies of the screening tests were clearly described. They also noted that the information was presented using short sentences and simple words that facilitated their understanding.

The web-based decision aid clearly compares the two screening tests and shows their advantages and disadvantages. All of their differences can be identified immediately. Before, if I needed to search for information, I had to search the tests one by one and then compare them by myself. This web-based decision aid shows all of their similarities and differences [together].(A17)

It is good because there are not many words, and the information is easy to understand. There is adequate information on the web-based decision aid, and it uses simple words to introduce the two screening tests’ risks and benefits, and similarities and differences.(A04)

The interviewees reported that they found the decision aid easy to use. They noted that the decision aid was well designed; used icons effectively and was visually appealing in terms of its colours, images and font size.

I think that the Web-based decision aid [is good because it] doesn’t have much text. It’s clear and attractive because of the colours and pictures. I read all of the items. Although I may not be able to recall all the content if you ask me about it, I was interested and thus read every item.(A06)

Every icon gives clear guidance and I can find what I want very quickly. This saves a lot of time. Furthermore, if you have any questions, you can ask them by pressing the WhatsApp button [“Talk to Nurse”]; this aspect is great.(A18)

(2) Informative and balanced content aided decision-making.

The interviewees found the Web-based decision aid to be informative and well-balanced in terms of the amount and content of information related to screening tests. They were able to access further information via links to additional resources (e.g. on where to obtain screening) to assist them in making screening decisions.

The amount of information provided is appropriate because it covers current screening methods such as the Pap test, and it also includes information about HPV test, which I don’t usually hear about. Now, I can learn more about these topics through the webpage.(A03)

The information on both sides [regarding screening] is unbiased and objective. It appears to be neutral…[so] it’s up to us to make our own choice…[we are]empowered to make our own decision.(A02)

(3) Facilitators of using the Web-based decision aid.

Generally, the interviewees did not encounter barriers to using the decision aid, and they reported several facilitators of its use. For example, it can be accessed conveniently from various devices with an Internet connection, and at their own pace and convenience. Some of the interviewees noted that being exposed to information in the media, in movies and from friends about cervical cancer increased their concerns about their own health and their awareness of the disease, thereby increasing their likelihood of using the decision aid. All of the participants revealed that reading the decision aid took approximately 10–30 min.

It’s not during working hours [that I use the decision aid]. When I am taking a break or taking a ride is when I will use it. I don’t have a fixed place to use it. If I have some free time, I can just grab my smartphone and browse the webpage.(A14)

I use it at home and usually after dinner at night.It takes about 20 minutes to read all of it.(A15)

(4) Areas for improvement.

On a computer, most of the information in the Web-based decision aid was compared and presented using either a vertical or side-by-side format. However, on a smartphone, the layout might have changed, as the information might have been presented vertically to fit the screen. Three participants suggested that tables should be added to facilitate reading on a smartphone.

I think it is important to have a table. When we scroll down the webpage on a smartphone, for example, the benefits and risks are shown one by one. This means that they are difficult to compare, unlike on a computer with a big monitor displaying all the information at once and vertically, which makes comparison easy.(A16)

Although some of the interviewees thought that the information in the decision aid was presented using short sentences and simple words, several interviewees commented that there were too many words. They suggested that to enhance the attractiveness of the decision aid, videos or motion graphics could be used to provide dynamic and interactive content.

More graphics could be added. If there were some short videos, [the decision aid] would be more attractive.(A07)

For example, in terms of information presentation, you could add some motion graphics that would increase people’s interest.(A01)

Two participants suggested that regular ‘booster’ notifications, such as screening reminders or updated news about screening tests, should be issued to keep users motivated and engaged. Four participants stated that more practical information and support, such as on the cost of screening, follow-up services available after screening and a person for them to talk to when they encounter difficulties in making the screening decision, should be provided.

There could be more interactive features, such as chatbots. After you ask your first question, these could tell you what you may need to know. This could be directional, that is, it could tell me, “Well, it may be a good idea for you to have this screening test.” This would make it clearer for me.(A17)

## Discussion

This pilot RCT assessed the feasibility and acceptability of a Web-based decision aid on cervical cancer screening for working women aged 25–44, and preliminarily examined its effects on selected outcomes. The rate of consent to participate was similar to that in a previous study on a decision aid for cervical cancer screening among South Asian women (80%) [15] and was higher than that in a previous study on a decision aid on HPV vaccination among Chinese parents (68%) [24]. The intervention and follow-up assessment completion rate in the current study was lower than that in one study (100%) [15] but higher than that in another study (83.2%) [24]. These differences could be due to between-study difference in the types of participants and the strategies used to monitor the use of a decision aid. Our participants were Chinese working women aged 25–44, whereas those in the first of the abovementioned studies were South Asian women aged 25–64, more than half of whom were not in paid employment [15]. Such women could make use of their spare time to read a decision aid after they have completed household chores and when their children are at school [15, 26]. In the current study, a follow-up telephone call was made to the intervention group to review whether they had read the decision aid and whether they had encountered any difficulties, but such follow-up support was not offered in the second of the abovementioned studies [24]. A follow-up call is useful, as it serves as a reminder for people who have not read a decision aid and enables support to be provided as necessary [15].

User acceptability could affect the utilisation of materials. Although not all of the intervention group had undergone screening, they appreciated that the Web-based decision aid could resolve decisional conflict, consistent with the finding of a previous study on a Web-based decision aid that aimed to promote breast cancer screening [26]. Such Web-based interventions allow users to receive information conveniently without time and place restrictions, with user-friendly designs and informative, balanced content about cervical cancer screening that gives users the autonomy to make their own decisions [15, 26]. The intervention group could read the entire decision aid within 10–30 min, which is less than the reading time reported for a decision aid on breast cancer screening [27]. A sufficiently brief reading time ensures that using the decision aid is not a burden to women and thus can support its utilisation. In this study, the interviewees provided valuable comments on how the Web-based decision aid could be further refined to enhance its attractiveness and usefulness. Thus, it should be refined based on these comments, such as by adding videos and motion graphics, and providing regular notifications, and the refined Web-based decision aid should then be tested in a full-scale RCT.

Consistent with previous studies, improvements in decisional conflict and risk perceptions were observed among the intervention group after they had used the decision aid [12, 27]. Their decreased overall decisional conflict score indicated that they were more ready to make a decision than they had been before using the decision aid. Some interviewees noted that they had thought that the Pap test was the major test used for cervical cancer screening and had learned from the decision aid that the HPV test was an alternative type of test. Thus, they felt that the decision aid had informed them about the screening choices available and therefore they could choose which screening to seek [15, 28]. Moreover, some interviewees noted that the decision aid had encouraged them to ask their doctors for an HPV test at their next visit [28]. Women’s attitudes on and perceptions of screening affect their screening behaviour [8, 9]. A values-clarification exercise helps women to review their values and thoughts about the importance of health maintenance in their life [13]. In the current study, the intervention group’s perception of having a high risk of developing cervical cancer increased after the intervention, although the magnitude of this increase was not as large as that reported in a previous study conducted among South Asian women [15]. This difference may be attributable to the fact that 41% of the intervention group in the current study had received an HPV vaccination, whereas none of the South Asian women in the previous study had received an HPV vaccination [15]. That is, women who have received an HPV vaccination may think that this protects them from cervical cancer and thus regard themselves as having a lower risk of developing cervical cancer than those who have not received an HPV vaccination [28].

Interestingly, although 32% of the intervention group had undergone cervical cancer screening, this was a lower uptake rate than that reported in a previous study conducted among South Asian women, i.e. a 62.5% uptake rate [15]. This difference may be attributable to the employment statuses of the participants in each study, as this could limit women’s choice in appointment scheduling. In the current study, a link to information about available resources to support screening (e.g. screening service providers) was provided in the decision aid, but the users had to book a screening service appointment themselves. In contrast, in the abovementioned previous study, South Asian women could seek support from community health workers to schedule an appointment [15, 29]. Some of the interviewees in the current study revealed that they lacked proficiency in following telephone instructions, which is required for making screening appointments in government-run clinics. Thus, they hope to have further support in this appointment booking process [28]. Furthermore, during the COVID-19 pandemic, some government-run clinics suspended their services [3], meaning that some interviewees had been unable to book appointments near their homes and thus had had to consider making appointments at clinics far from where they lived or that were more expensive than their local clinics. Given that approximately 30% of the women in this study had a monthly income of less than HKD20,000, cost would be one of their considerations in making a decision about screening [3, 8]. A high cost decreased their motivation to undergo screening, and some interviewees noted that they would undergo screening when their local screening services resumed.

There are some limitations to this study that must be acknowledged. First, the sample was recruited via convenience sampling and from our social network. Thus, there might have been selection bias that threatened the representativeness of the sample. Second, it was impossible to blind the staff to whether they were helping to deliver the decision aid or the control treatment. Third, screening uptake by the intervention group was determined at the 3-month follow-up, but some of the intervention group might have scheduled their appointment after this time. Thus, a longer follow-up period, such as 6 months, should be adopted in a future full-scale study. Fourth, Web analytics like number of page views or amount of time spent viewing a page were not collected. These should be collected in future studies as they may provide more information about how the decision aid is used.

## Implications for Practice and Research

The Web-based decision aid may be a useful tool to support and allow young working women to take an active role in deciding whether to undergo cervical cancer screening. In addition, as the decision aid can help promote the need to undergo screening to young working women, it will be revised based on the comments received from the interviewees in the current study. Additional strategies such as provision of navigation support, may be adopted to overcome the barriers experienced by those women who have not undergone screening. In addition, we hope that it can be extended for use by women aged 45–64, such that more women will undergo cervical cancer screening than is currently the case. A full-scale RCT targeting women aged 25–64 years is essential to evaluate the ability of our refined decision aid with additional navigation support provided to enhance the timeliness of cervical cancer screening uptake by women who are eligible for such screening.

## Conclusion

The implementation of a Web-based decision aid was feasible and acceptable among a group of young working women. The preliminary findings suggest that the decision aid can help to reduce decisional conflicts, clarify values, improve risk perception and encourage cervical cancer screening uptake. Full-scale studies are needed to confirm its effects.

## Supplementary Information

Below is the link to the electronic supplementary material.

ESM 1 (DOC 218 KB)

ESM 2 (DOCX 1.18 MB)

## Data Availability

The data that support the findings of this study are available from the authors upon reasonable request.
