# Supplementary material for: Effects of Web-Based Decision Aid to Support Cervical Cancer Screening Decision Among Young Working Women: A Pilot Randomised Controlled Trial
Source: Int J Behav Med. 2024 Dec 30;33(1):56–68. doi: 10.1007/s12529-024-10344-9 (PMC12935772; doi:10.1007/s12529-024-10344-9)

**Supplementary material: Screen capture of the web-based decision aid**

1. Main page


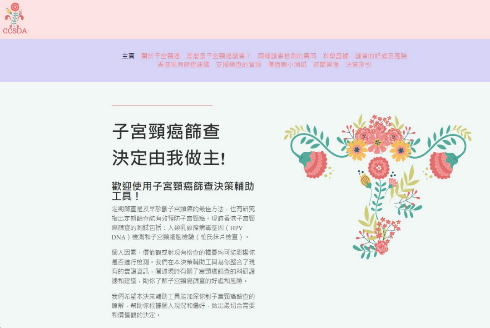


1. Information about cervical cancer


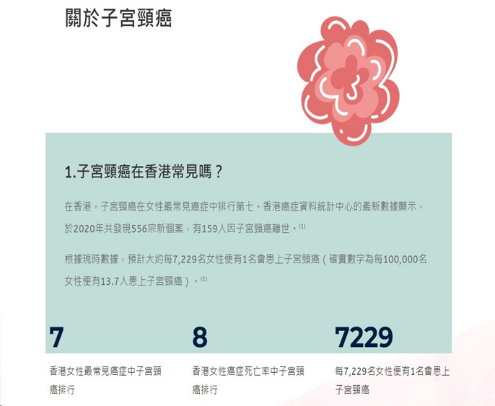


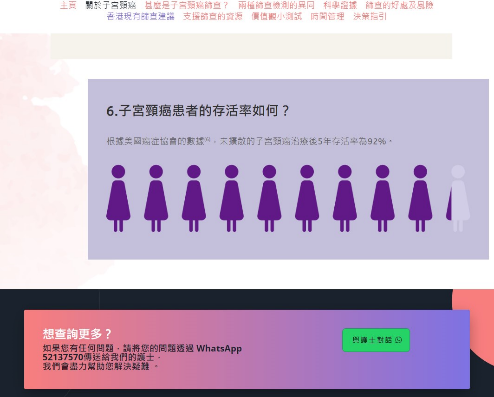


1. Available screening options and comparison of similarities and differences


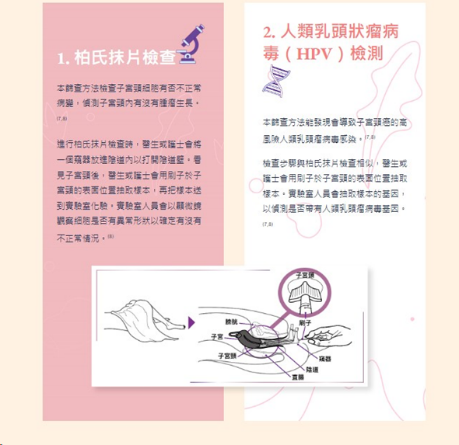

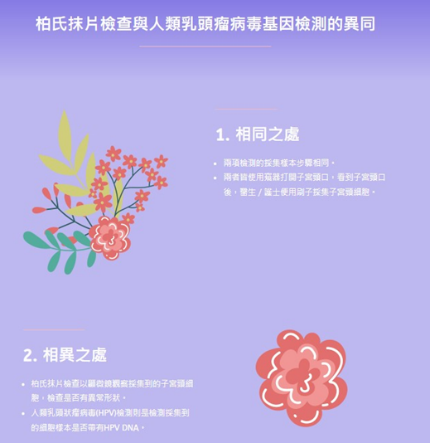


1. Accuracy of screening options and current screening recommendations


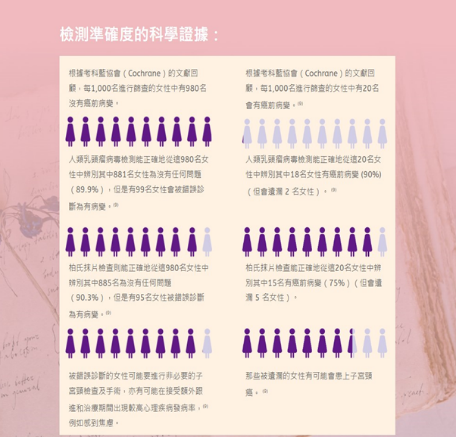

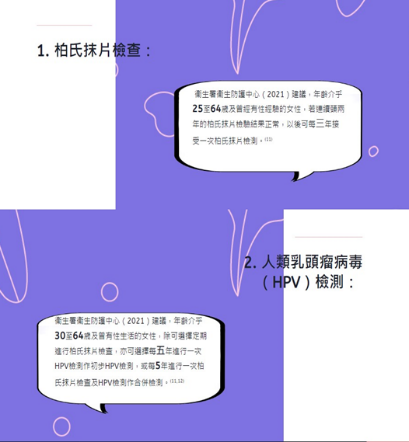


1. Value clarification exercise


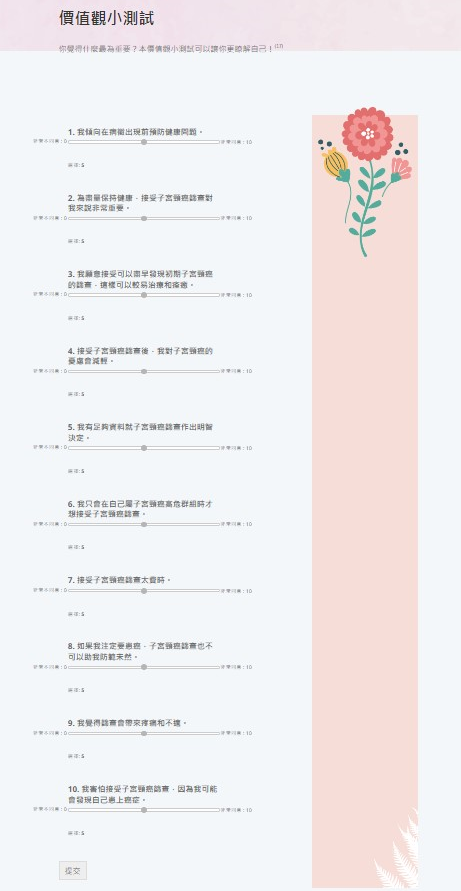

Supplement: Supplementary file 2 — (DOCX 1.18 MB) [file 12529_2024_10344_MOESM2_ESM.docx]
